# Oxygen: viral friend or foe?

**DOI:** 10.1186/s12985-020-01374-2

**Published:** 2020-07-27

**Authors:** Esther Shuyi Gan, Eng Eong Ooi

**Affiliations:** 1 Programme in Emerging Infectious Diseases, Duke-NUS Medical School, Singapore, Singapore; 2 Saw Swee Hock School of Public Health, National University of Singapore, Singapore, Singapore; 3 Department of Microbiology and Immunology, National University of Singapore, Singapore, Singapore

**Keywords:** Hypoxia, Viruses

## Abstract

The oxygen levels organ and tissue microenvironments vary depending on the distance of their vasculature from the left ventricle of the heart. For instance, the oxygen levels of lymph nodes and the spleen are significantly lower than that in atmospheric air. Cellular detection of oxygen and their response to low oxygen levels can exert a significant impact on virus infection. Generally, viruses that naturally infect well-oxygenated organs are less able to infect cells under hypoxic conditions. Conversely, viruses that infect organs under lower oxygen tensions thrive under hypoxic conditions. This suggests that in vitro experiments performed exclusively under atmospheric conditions ignores oxygen-induced modifications in both host and viral responses. Here, we review the mechanisms of how cells adapt to low oxygen tensions and its impact on viral infections. With growing evidence supporting the role of oxygen microenvironments in viral infections, this review highlights the importance of factoring oxygen concentrations into in vitro assay conditions. Bridging the gap between in vitro and in vivo oxygen tensions would allow for more physiologically representative insights into viral pathogenesis.

## Background

Viral infections are heavily dependent on host cells for energy, enzymes and metabolic intermediates for successful replication [134]. Factors that influence the state of a cell, including differential gene expression and pathway activation, could all impact the outcome of viral pathogenesis. One such factor is the oxygen level in the microenvironment in which cells reside. Oxygen plays key roles in respiration, metabolism and energy production. Given the key role of oxygen in cell function, cells have evolved oxygen sensors that regulate the expression of a suite of genes in response to lowered oxygen levels. For this discovery, William Kaelin, Gregg Semenza and Peter Ratcliffe were awarded the 2019 Nobel Prize for Physiology and Medicine. Here, we elaborate upon our understanding of how cells react to different oxygen levels and review how oxygen affects the outcome of viral infection and disease pathogenesis. In general, viruses that naturally infect and replicate in tissues with high oxygen content are impaired by hypoxic environments. Conversely, hypoxia has been shown to increase the infection of viruses that naturally infect organs with lower oxygen tensions.

## Main text

### Oxygen cascade

Although oxygen is needed by all human cells, not all cells in our bodies receive similar amounts of oxygen. Oxygen levels in most organs, with a few exceptions, are lower than that of atmospheric oxygen (20–21% or 152-160 mmHg). This disparity is largely due to blood transportation through the vascular anatomy and subsequently vascular beds in tissues [55]. Due to its poor solubility in liquids, oxygen is transported around the body by hemoglobin in red blood cells. Each hemoglobin molecule carries up to a maximum of 4 oxygen molecules with its affinity for each oxygen molecule increasing as each of its binding sites is occupied [120]. Oxygen delivery in the human respiratory system depends on several factors such as the partial pressure of oxygen, efficiency of gas exchange, concentration and affinity of hemoglobin to oxygen and cardiac output [92]. The highest oxygen concentration is typically found in the respiratory tract. As respired air enters the trachea and is humidified in the upper respiratory tract, the pressure of oxygen decreases while concentration of water increases, thus altering the partial pressure of oxygen in this gas mixture [92]. Further dilution occurs as oxygen diffuses in and out of arteries. This is best exemplified in organs such as the spleen and liver. In spleens, oxygen concentrations are highest nearest the splenic artery (~ 6%) as compared to locations in the spleen distant from the splenic artery (~ 1%) [16]. In the liver, oxygen tensions range from approximately 12% oxygen surrounding the portal vein to 1% oxygen in the proximity of the central vein [137]. The average oxygen concentrations of different organs observed in humans and animal models are summarized in Table 1. Taken collectively, with the exception of the lungs which are exposed to ambient air, median physiological oxygen tensions of organs are significantly lower than that of atmospheric oxygen tensions. This is known as the oxygen cascade. Thus, physiological oxygen concentrations in which viral infection and replication occur can be significantly different to the level of oxygen in normal air. It is therefore useful to understand how cells adapt to physiological oxygen tension.

**Table 1:** Summary of oxygen concentrations in various organs

Tissue	O_2_ (%)	mmHg	Species	Reference
Atmospheric Air	21.1	160	Human	[17]
Trachea	19.7	150	Human	[17]
Arterial Blood	13.2	100	Human	[17]
Venous Blood	5.3	40	Human	[17]
Brain	4.4 ± 0.3	33.8 ± 2.6	Human	[4, 102]
Normal Lung	5.6	42.8	Human	[93]
Lung Tumor	0.1–6.1	0.7–46	Human	[93]
Skin (Epidermis)	1.1 ± 0.42	8 ± 3.2	Human	[147]
Skin (Dermal Papillae)	3.15 ± 0.8	24 ± 6.4	Human	[147]
Liver	7.5 ± 0.7	40.6 ± 5.4	Human	[13, 94]
Kidney	6.8 ± 0.8	52 ± 6	Human	[108]
Kidney	5.9–6.6	45–50	Rat	[129]
Placenta	7.4 ± 0.4	56.2 ± 3.2	Human	[76]
Umbilical cord	2.7–3.9	20–30	Human	[47]
Umbilical artery	1.3–1.9	10–15	Human	[47]
Bone Marrow	7.22 ± 0.1	54.9 ± 0.98	Human	[60]
Ovaries	11.6	88	Human	[43]
Spleen	10 ± 2.4	80 ± 18	Rats	[65]
Lymphoid organs	0.5–4.5	3.8–34.2	Mice	[16]
Skeletal muscle	3.3 ± 0.58	25 ± 4.4	Human	[8]
Adipose tissue	4.7–8.9	36–68	Human	[40]

### The HIF family and molecular mechanisms of oxygen sensing

In microenvironments with lowered oxygen levels, cells regulate the expression of genes, such as those involved in controlling angiogenesis, iron metabolism and glycolysis, to adapt and survive. To understand the cellular response to lowered oxygen levels, investigators focused on the regulation of erythropoietin (EPO), that is known to be induced in response to lowered oxygen to stimulate erythropoiesis. Analysis of the cis-acting sequences involved in EPO induction led to the identification of hypoxia inducible factor (HIF) [130, 143, 144].

### The HIF family

HIF transcription factors are basic helix-loop-helix DNA binding proteins of the PER-ARNT-SIM family [143]. HIFs form heterodimers, where alpha subunits HIF1α, HIF2α, HIF3α [39, 49, 130] interact with a constitutively expressed beta subunit HIF1β, also known as the aryl hydrocarbon receptor nuclear translocator 1 (ARNT1) [145]. HIF1α and HIF2α are oxygen sensitive subunits that share 48% genetic sequence homology [63]. Both dimerize with HIF1β during hypoxic conditions to induce gene transcription [39]. HIF3α is distantly related, sharing less sequence homology and function with HIF1α or HIF2α. It has 6 splice variants [49]. Its function remains understudied in comparison to HIF1α and HIF2α although in vitro studies suggest that the prevailing actions of HIF3α variants are inhibitory and constitutes a negative feedback loop for HIF1α and HIF2α [59, 98].

Both HIF1α and HIF2α proteins have multiple conserved domains involved in DNA binding, protein interaction and dimerization, oxygen-dependent degradation (ODD) and transcriptional activity (N-TAD and C-TAD). HIF3α isoforms are shorter and carry only a N-TAD domain together with a leucine zipper motif with unknown function [99, 115]. HIF1β contains no transcriptional activation domains and requires dimerization with HIF1α to induce transcription. With 70, 85 and 100% homology between their basic helix-loop-helix DNA binding and remaining basic domains, it is not surprising that HIF1α and HIF2α binds DNA indistinguishably [136]. While extremely similar in both homology and function, there are subtle differences between HIF1α and HIF2α. The C-TAD domains of HIF1α and HIF2α control target gene transcription through the recruitment of co-factors but target gene selectivity between the 2 proteins have been postulated to arise from the N-TAD domains which recognize distinct transcriptional co- factors [3, 32, 38, 67]. Besides differences in protein domains, the expression of this protein is variable in different cell types. HIF1α is expressed in almost all immune cell types including neutrophils [142], monocytes [12, 131], macrophages [29], dendritic cells [11, 75] and lymphocytes [9, 101]. HIF2α however is only expressed in certain cell types such as endothelial cells [66] and tumor associated macrophages [72].

Immune cells in the circulatory system are exposed to a gradient of oxygen concentrations in the blood, lymphoid organs and areas of inflammation [120, 121]. It is therefore essential for immune cells such as monocytes, macrophages and dendritic cells to rapidly adapt to fluctuating oxygen. HIF1α has indeed been implicated in all facets of the immune response including inflammation [113], responses to bacterial and viral infections [46, 104, 126, 135], immune cell metabolism [28, 103, 111] and lymphoid cell development [19]. HIF1α could thus be a major regulator of infection outcome.

### Oxygen sensing mechanisms of HIF1α

**Fig. 1:**
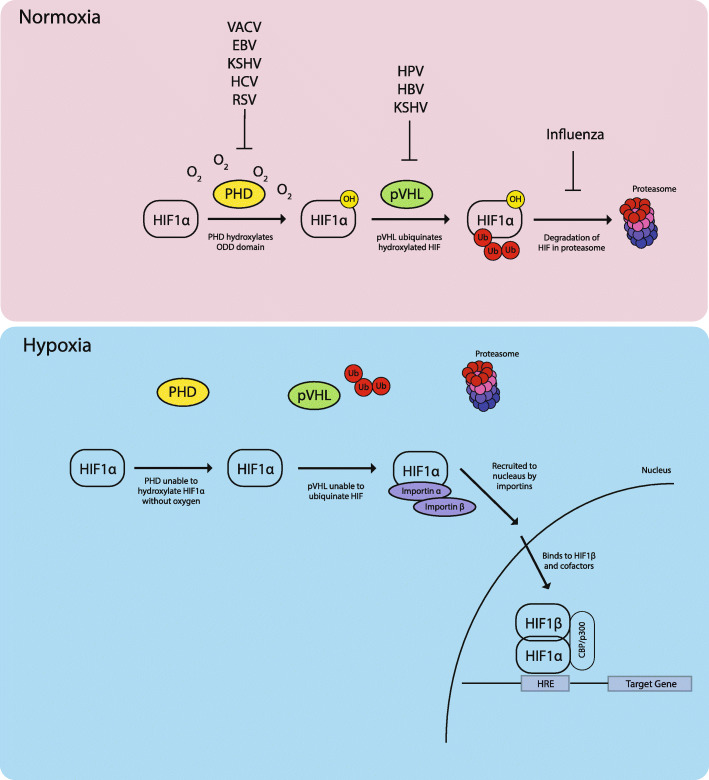
The oxygen sensing pathway of HIF1α. HIF is the master regulator of the cellular hypoxic response. Under normoxic conditions, HIF1α is hydroxylated in an oxygen dependent manner and tagged for degradation by VHL complexes. In low oxygen environments, HIF1α is stabilized due to the lack of oxygen, translocate to the nucleus and forms a heterodimer with HIF1β and other cofactors to activate transcription of hypoxia inducible genes. Viruses that are able to stabilize HIF1a under atmospheric conditions by inhibiting PHD or VHL interactions are shown in the top panel

HIF1α sensitivity to cellular oxygen tension is largely dependent on post-translational modifications. While HIF1α is constitutively expressed, it is highly regulated by oxygen and has a short half-life of approximately 5 mins [125]. Sufficient oxygen in the cellular environment, such as those in most in vitro experiments, will result in rapid proteasomal degradation of HIF1α in the cytoplasm [38]. An overview of these processes is shown in Fig. 1.

In oxygen rich environments, a family of prolyl hydroxylase domains (PHD1–3) hydroxylate highly conserved proline residues (Pro402 and Pro564) in the ODD domain of HIF1α in an iron-, α-ketogluterate-, ascorbate- and oxygen-dependent manner [12, 29]. This hydroxylation enables the binding of the von-hippel Lindeau (VHL) E3 ubiquitin ligase complex to HIF1α. Ubiquitination of HIF1α then initiates the process of proteasomal degradation. In addition to PHDs, an independent regulatory hydroxylation step by Factor Inhibiting HIF (FIH) hydroxylates an aspargine (Asn803) to interfere with HIF1α ability to recruit and bind to co-factors via its C-TAD domain [30, 44]. Additional negative regulation of HIF1α occurs by acetylation of Lys532 by acetyl transferase arrest-defective 1[77], GSK3β phosphorylation of Ser551, Ser558 and Ser559 [107] and PLK3 phosphorylation of Ser576 and Ser 659 [149]. All these processes contribute to HIF1α destabilization leading to proteasomal degradation of HIF1α, which prevents transcription of hypoxia inducible genes [77, 107, 149]. Clinically, patients with von Hippel-Lindau syndrome, where VHL is defective, have an overproduction of hypoxia-inducible genes encoding for angiogenesis leading to the development of multiple tumors [122].

As PHD and FIH hydroxylation of HIF1α is oxygen-dependent, any decrease in oxygen levels would lead to stabilization and nuclear translocation of HIF1α. This transport occurs when importin-α binds to the nuclear localization signal in the C-terminal NLS of HIF1α, recruit importin-β and initiates nuclear translocation [35]. In the nucleus, HIF1α forms heterodimers with HIF1β. It has been postulated that HIF1β preferentially binds to HIF1α phosphorylated by MAPK243, therefore increasing transcriptional activity of HIF1α. However transcriptional activity requires N-TAD and C-TAD domains to recruit co-factors CBP/p300, SRC-1 and TIF2 to induce efficient transcription of target genes [18, 91]. Through its interaction with CBP/p300, S-nitrosation on cysteine800 has also been shown to increase HIF1α transcription activity [151]. Other co-factors for HIF1α include pyruvate kinase M2 isoform [97] and mediator associated kinase CD8K [45], ATPase/helicase chromatin remodeling factor Pontin [95] and SWI/SNF nucleosome remodeling complex [82].

Transcription of hypoxia-inducible genes occurs when HIF1α/HIF1β heterodimer bind to a core consensus sequence 5′– (A/G) CGTG − 3′ within the hypoxia response element (HRE) at the proximal promoters of target genes [128]. Though the HRE sequence is abundant throughout the human genome, HIF1α only binds to approximately 1% of such sequence [128]. In addition to the HRE, several HIF1α target genes contain a HIF ancillary sequence (HAS) 5′-CAGGT-3′, which is an imperfect inverted repeat of the HRE. Kimura and colleagues have shown that alteration of this sequence affects the HIF1α-induced transcriptional activity of EPO [86, 87]. Interestingly, HIF1α preferentially binds HRE at regions of chromatin with DNaseI hypersensitivity, RNA polymerase II, basal transcriptional activity and histone modifications [105, 128, 148]. This may serve to explain why HIF1α has cell-type specificity in function.

### Hypoxia as a consideration for in vitro studies

Stabilization of HIF in cells residing in low oxygen microenvironments drives cellular reprogramming that also affects the availability of pro-viral and anti-viral host factors that collectively determine the outcome of viral infections. For instance, increase in glycolysis has been shown to be favorable for dengue virus (DENV) [41], herpes simplex virus (HSV) [1], human immunodeficiency virus (HIV) [62], rubella virus [7], hepatitis C virus (HCV) [80], influenza [133] and norovirus [116] infection.

A growing body of evidence suggests that, besides glycolysis, many other hypoxia-driven changes will also have important implications not only in the study of viral pathogenesis.

### Viruses which lifecycle is inhibited by hypoxia

Viruses that infect the respiratory tract are generally restricted by hypoxia [36]. This restriction potentially impacts the use of recombinant adenoviruses as a vector for cancer vaccine. Hypoxia is an important feature of solid tumors and the ability of oncolytic viruses such as adenoviruses or vesicular stomatitis virus (VSV) to replicate under these low oxygen conditions could be a critical determinant to the success of these therapies. However, in vitro, significantly reduced synthesis of adenovirus (wildtype strain Ad309) viral protein was observed when cells were cultured at 1% oxygen, ultimately leading to lower production of infectious Ad309 compared to experiments in normal air [119]. As tumor hypoxia has been shown (8-10 mmHg) [64], this restriction of adenovirus infection could be a factor in poor intratumoral spread resulting in the limited efficacy observed in clinical trials [150].

Perhaps a more promising oncolytic virus is VSV, which has been shown to overcome hypoxia-induced restrictions in viral protein translation during early infection in vitro [27]. However, renal carcinoma cells (RCC) lacking pVHL, resulting in constitutive HIF activity, showed greater resistance to the cytolytic effect of VSV as compared to wildtype RCC. Gene expression profiling under these conditions indicates that HIF enhances IFNβ upon VSV infection. This suggests that HIF could play an antiviral response against VSV, and should be an important consideration when used as an oncolytic viral therapy [70] (Table 2).

**Table 2:** Summary of viruses inhibited and augmented by hypoxia

	Mechanism	Cell Line	Conditions	Reference
Inhibited				
Adenovirus	Cell arrest	H1299 / A549	1% oxygen	[119, 132]
SV40	Blocks replication	CV1	0.02% oxygen	[124]
H1 parvovirus	ND		Constitutive expression of HIF1α	[25]
VSV	Increase in innate immune responses	RCC	Constitutive expression of HIF1α	[69]
Augmented				
DENV	Increased antibody dependent uptake via upregulation of FcγRIIA and membrane ether lipid concentrations	THP-1, Primary Monocytes	3% oxygen	[46]
	Correlated with increased anaerobic glycolysis for increased ATP production	Huh7	3% oxygen	[42]
EBV	Induce reactivation of EBV	Burkitt lymphoma Sal	Deferoxamine (DFX)	[90]
HSV	Hypoxia induced GADD34	U87	5% oxygen	[2]
HCV	ATP increase due to the induction of anaerobic glycolysis	Huh7.5	3% oxygen	[139]
Sendai Virus	ND	Rhabdomyosarcoma	3 Kpa	[37]
KSHV	HIF1α induction of viral Rta promoter	Hep3B	Expression of reporter plasmids containing Rta promoter	[58]

*ND* Not determined

### Viruses enhanced by hypoxia

On the other hand, a multitude of viruses replicate in organs with oxygen microenvironments significantly lower than that of atmospheric air. To establish successful infection, these pathogens would have evolved to thrive in cells adapted to such oxygen microenvironments. Indeed, low oxygen levels have been shown to be advantageous to a number of viruses (Table 1). Under hypoxic conditions, cells develop a metabolic response to ensure their survival, in part by upregulating anaerobic glycolysis for energy production In hepatocytes, this increase in anaerobic glycolysis directly correlated with increases in DENV replication [42]. Similarly, hypoxia enhances the replication and promotes a sustained infection of hepatitis C virus (HCV) by triggering alterations in liver cellular bioenergetics resulting in a higher rate of anaerobic glycolysis in a HIF-independent manner [139].

In addition, as cells adapt to a lack of oxygen, a multitude of lipids, proteins and signaling pathways are differentially expressed, which can be potentially advantageous for viral infection. For example, enhanced replication of herpes simplex virus (HSV) G207 under hypoxic conditions [2] is postulated to be due to hypoxia mediated upregulation of GADD34, which complements the replication of HSVs deficient in the viral gene γ34.5 [61]. Similarly, hypoxia enhances parvovirus B19 replication [15, 24, 118] by upregulating cellular Epo/EpoR receptor signaling in erythroid progenitor cells (EPCs) [24] which have been shown to be vital for parvovirus B19 replication [23]. This could be a contributory factor to the specificity of parvovirus B19 for infecting EPCs, which reside in the bone marrow that has oxygen concentrations of 0–4% [114].

Another virus, Kaposi’s sarcoma-associated herpesvirus (KSHV) was the first virus identified to have a functional HRE in its Rta gene. As activation of Rta results in induction of the lytic replication of the virus, this suggests that hypoxia can directly stimulate KSHV replication via HIF1α [58, 140]. Indeed, it has previously been shown that hypoxia induces the lytic replication of KSHV in primary effusion lymphoma cell lines [31].

More recently, dengue virus (DENV) infection and replication has been shown to be enhanced in monocytes at oxygen levels comparable to that within the lymph nodes (3%). This enhancement was observed both in a context of a DENV-only infection as well as antibody-dependent infection that simulates clinical secondary infection with a DENV serotype heterologous to the primary infection [52–54, 57]. DENV exists as 4 antigenically distinct serotypes. Antibodies produced after infection with one DENV serotype are able to enhance infection with the remaining 3 serotypes. Binding of cross-reactive or sub-neutralizing levels of antibodies to DENV enables viral entry into myeloid-derived cells via the fragment crystallizable gamma receptor (FcγR) [5, 10, 20–22, 26, 33, 146]. This route of infection is also commonly referred to as antibody dependent enhancement (ADE). When monocytes are incubated at 3% oxygen, HIF1α directly binds to and upregulates transcription of FcγRIIA. Moreover, hypoxia-dependent but HIF1α-independent changes in cellular membrane lipid composition further complement the increase in FcγRIIA to increase uptake of antibody-opsonized DENV. This synergistic effect is attributed to the increased proportion of ether phosphatidylethanolamine (ether-PE) in membranes of cells cultured under hypoxic conditions [46]. Taken together, such hypoxia induced changes increase myeloid cells susceptibility to antibody-dependent DENV infection. It also suggests that assessment of virus neutralizing antibodies should be conducted in myeloid cells incubated at oxygen levels that reflect the microenvironment of the lymph nodes, where these cells reside and function.

### Viruses that stabilize HIF1α in an oxygen independent manner

Besides relying on the level of oxygen to modulate host cell responses via the HIF pathway, some viruses have evolved the ability to interact with components of this pathway for its benefit. Pathogens such as influenza virus [123], vaccinia virus (VACV) [100], Epstein-Barr virus (EBV) [141] and hepatitis B virus (HBV) [153] have been shown to stabilize HIF1α after infection to stimulate the transcription of hypoxia inducible genes, even under normoxic conditions (Fig. 1, Table 3).

**Table 3:** Viruses that induces a pseudohypoxic state

Induces Hypoxia Response	Effect	Mechanism	Cell Line	Reference
Influenza	HIF1α stabalization	Impaired proteasome function results in decreased degradation of HIF1α	A549	[123]
Hepatitis B Virus (HBV)	HIF1α stabalization	HBx inhibits binding of VHL to HIF2α	HepG2, L02	[68]
	HIF1α stabalization	HBx activates MAPK pathway which in turns induces the activity of HIF1α	Chang X-34, HepG2	[153]
VACV	HIF1α stabalization	C16 inhibition of PHD2	Hek293T	[100]
EBV	Increased protein synthesis of HIF1α	LMP induced	KR-4	[141]

One strategy employed by viruses to stabilize HIF is to inhibit its interaction with PHDs and VHLs (Fig. 1). VACV protein C16 stabilizes HIF1α by directly binding to PHD2 and inhibiting hydroxylation and subsequent degradation of HIF1α. This results in rapid stabilization of HIF1α early post infection activation of HIF1α response genes [100]. HIF1α is similarly stabilized during EBV infection indirectly via latent membrane protein 1 (LMP1), inducing proteasome degradation of PHD1 and 3. As a result, LMPs prevents the formation of HIF/VHL complexes which are required for HIF1a degradation [89, 141]. Importantly, as HIF1α activation induces angiogenesis, its stabilization during EBV infection may play important roles in EBV-mediated tumorigenesis and tumor progression. In another example, KSHV employs several strategies to stabilize HIF1a such as the expression of a miRNA cluster within the viral genome that binds PHD1 mRNA to downregulate its expression. In addition, the KSHV protein LANA targets VHL for degradation [14, 152]. Together, these result in the stabilization and increased activity of HIF1α during KSHV infection.

Viruses such as HCV and RSV are also known to reprogram cellular metabolism to stabilize HIF1α under normoxic conditions. Oxidative stress induced by HCV infection results in HCV-stabilized HIF1α which subsequently leads to synthesis and secretion of VEGF [109]. Similarly, RSV infection in bronchial airway epithelial cells induce the release of nitric oxide (NO), which results in HIF1α stabilization and expression of HIF1α target genes [84]. This is likely due to increased oxygen consumption via oxidative phosphorylation, which results in redistribution of intracellular oxygen away from PHDs to respiratory enzymes, so that the cell senses internal hypoxia [56].

Similarly, viruses that attenuate the VHL and HIF1a interaction results in the induction of the hypoxic response. During HBV infection, the HBV X protein (HBx) increases the expression, stabilization and transcriptional activity of HIF1α by binding to and inhibiting their interaction with VHL [68, 96, 106, 153]. In HPV infections, the oncoprotein E6 forms a complex with HIF1a to inhibit its association with VHL and thus protect HIF1a from proteasome dependent degradation [51]. This directly leads to HIF1α induced glycolysis, which contributes to the Warburg effect seen in cancer cells.

Although influenza virus naturally infects respiratory epithelial cells that are exposed to atmospheric conditions (20–21% O_2_ or 152–160 mmHg), recent studies suggest that H1N1 virus infection can trigger a hypoxic response. Under normoxic conditions, H1N1 influenza virus infection stabilizes HIF1α by inhibiting proteasomal activity, resulting in the activation of the HIF1α pathway [123]. Nuclear accumulation of HIF1α resulted in enhanced proinflammatory cytokines secreted from infected cells that could thus play a role in the development of severe inflammation during H1N1 infection [50].

### HIF1α inhibitor as a potential anti-viral strategy

The identification of the role of HIF1α in viral infection suggests a unique opportunity for HIF1α inhibitors to be used as anti-viral drugs. HIF1α inhibitors may be effective against viral infections that have exhibited the ability to induce HIF1 α and thrive under its activity. To date, HIF1α inhibitors have been developed primarily for cancer tumor therapy. These inhibitors act on various processes [112], including HIF1α mRNA expression, protein translation, protein degradation, DNA binding and transcriptional activity (Table 4).

**Table 4:** Summary of HIF1α inhibitors

Mode of inhibition	Compound	Target	Reference
HIF1α mRNA	EZN-2968	HIF1α	[117]
HIF1α Translation	Topotecan / AZN-2208	Topoisomerase 1	[6, 127]
	PX-478	HIF1 / HIF2 Protein	[88]
	Digoxin	HIF1α protein	[156]
	Temisirolimus / Everolimus / MLN0128 / Metformin	mTOR	[73, 157]
	Wortmannin	PI3K	[155]
HIF1α Degradation	17-AAG / 17-DMAG	HSP90	[110]
	Romidepsin / Trichostatin	HDAC	[71, 81]
	LW6	HDAC / VHL	[85]
HIF1α DNA Binding	DJ12	HRE	[79]
HIF1α transcriptional activity	Chetomin	CH1 domain pf p300	[83]

While an increase in mRNA expression does not necessarily equate an increase in protein expression, HIF1α mRNA levels have been suggested to be a rate-limiting factor for its translation [154]. A highly specific RNA antagonist EZN-2968 is an antisense oligonucleotide that has been shown to bind to HIF1α mRNA to reduce its translation both in vitro and in vivo. This then resulted in the reduction of HIF1α transcriptional targets [48]. Phase I clinical trials showed safety in patients and further studies are required to investigate EZN-2968 modulation of HIF1α transcriptional targets [78, 117].

To inhibit HIF1α protein translation and accumulation in cells, agents such as inhibitors of topoisomerases I and II, receptor tyrosine kinase, cyclin dependent kinases and signaling pathways are all possible candidates. As studies have shown that mTOR plays a role in HIF1α translation, inhibiting mTOR signaling could lead to downregulation of HIF1α. Indeed mTOR inhibitors such as temsirolimus [34], everolimus [138], metformin and MLN0128 [73] have all been shown to inhibit HIF1α protein translation. Stabilization of HIF1α in the cytosol requires interaction with the chaperone protein HSP90. In the presence of HSP90 inhibitors, HIF1α undergoes proteasomal degradation [74]. Therefore HSP90 inhibitors such as 17-AAG and 17-DMAG are currently in development for cancer therapy [110]. In addition, small molecules may also downregulate the activity of HIF1α by inhibiting its ability to bind to HRE and initiate transcription of target genes. Recently, a compound DJ12 was identified from a screen of 15,000 compounds to inhibit HIF1α activity by blocking its binding to HRE sequences [79]. The development of such HIF1α inhibitors provide unique and hitherto unexplored opportunities to expand our anti-viral pharmacopoeia.

## Conclusion

Collectively, differences between atmospheric oxygen tensions in which in vitro experiments are generally conducted in differs drastically from that of physiological tissue oxygen microenvironments. In light of the growing body of evidence on the relationship between oxygen tensions and viral replication, the application of tissue oxygen tensions should be an important consideration when studying viral pathogenesis.

## Data Availability

Not applicable.

## Abbreviations

EPO: Erythropoietin
HIF: Hypoxia Inducible Factor
ARNT1: Aryl hydrocarbon receptor nuclear transporter 1
ODD: Oxygen dependent degradation
VHL: Von-hippel lindeau
PHD: Prolyl hydroxylase domains
FIH: Factor inhibiting HIF
PMK2: Pyruvate kinase M2 isoform
HRE: Hypoxia response element
HAS: Hypoxia ancillary sequene
DENV: Dengue virus
HSV: Herpes simplex virus
HIV: Human immunodeficiency virus
HCV: Hepatitis C virus
VSV: Vesicular stomatitis virus
KSHV: Kaposi’s sarcoma-associated herpesvirus
ADE: Antibody dependent enhancement
RCC: Renal carcinoma cells
VACV: Vaccinia virus
DFO: Deferoxamine
